# P04-13 How actively older persons age in Italy?

**DOI:** 10.1093/eurpub/ckac095.067

**Published:** 2022-08-29

**Authors:** Benedetta Contoli, Valentina Possenti, Valentina Minardi, Nicoletta Bertozzi, Giuliano Carrozzi, Marco Cristofori, Amalia Maria Carmela De Luca, Pirous Fateh-Moghadam, Mauro Ramigni, Massimo Oddone Trinito, Maria Masocco

**Affiliations:** 1 National Centre for Disease Prevention and Health Promotion, Istituto Superiore di Sanità, Rome, Italy; 2 Department of Public Health, LHU Romagna, Cesena, Cesena, Italy; 3 Department of Public Health, LHU Modena, Modena, Italy; 4 Operative Unit for Surveillance and Health Promotion, LHU Umbria 2, Orvieto, Orvieto, Italy; 5 DG Health, LHU Catanzaro, Catanzaro, Italy; 6 Department of Prevention, LHU Trento, Trento, Italy; 7 Department of Prevention, LHU 2 Marca Trevigiana, Treviso, Treviso, Italy; 8 Department of Prevention, LHU Rome 2, Rome, Italy

**Keywords:** Elderly, behavioural surveillance, healthy ageing

## Abstract

**Background:**

One scope of the Active and Healthy Ageing framework is to increase awareness on elderly-related topics. The year 2020 has seen an upheaval across the world caused by the COVID-19 emergence, even higher to older persons.

**Methods:**

In Italy, data gathered by the PASSI d'Argento behavioural surveillance system on general population aged 65+ in the timeframe 2016-2019 describe health conditions, lifestyles and care needs for elderly.

**Results:**

Basing on physical activity recommended by the WHO globally, 33% of non-physically impaired older persons reaches out those levels, 27% are partially active, 40% is sedentary. 9% fell down within 30 days prior the interview, accessing hospital was necessary in 19% of cases; 64% of falling occurred at home, 20% outdoor. 61% refers at least one infrastructural housing issue, 15% perceive higher neighbourhood insecurity. 35% reported difficulties in accessing essential services, especially to local health premises and for necessities. About 19% lives socially isolated, 21% had not any contact (neither by phone) with anyone in a typical week, 71% do not attend collective meetings, such as at a club or church. Nearby 1 out of 3 (29%) represents an asset to the own family/community: 19% looks after cohabiting people, 14% relatives or friends not living together with, 6% engage in volunteering. Participation in training courses or social events (trips/stays organised) regards little more than 2 over65 out of 10: 5% partakes in learning courses, 23% enjoyed those latest occasions. Such low social connectedness is observed even among «younger elderly» (aged 65-74). Nearly 19% referred frailty impacting on their own families mainly, 94% of frail elderly is given help from relatives, 20% from professional caregivers, 12% from acquaintances. All these factors suffer from socio-economic and territorial differences. Among elderly reporting many economic difficulties, social isolation is 31%, frailty 28%, difficult access to services 58%, falling 15%, with a geographic gradient at the expense of the Southern Regions.

**Conclusions:**

COVID-19 is a clear threat to older persons: in Italy, monitoring ageing dimensions under the pandemic scenario represents even a greater opportunity to have scientific data which describe the impact of health emergency on elderly.

